# Supplementary material for: Deletion of adipocyte Sine Oculis Homeobox Homolog 1 prevents lipolysis and attenuates skin fibrosis
Source: bioRxiv. 2024 May 22:2024.05.22.595271. Preprint. [Version 1] doi: 10.1101/2024.05.22.595271 (PMC11142148; doi:10.1101/2024.05.22.595271)
Supplement: Supplementary file 1 [file NIHPP2024.05.22.595271v1-supplement-1.pdf]

bioRxiv preprint doi: https://doi.org/10.1101/2024.05.22.595271; this version posted May 22, 2024. The copyright holder for this preprint (which was not certified by peer review) is the author/funder, who has granted bioRxiv a license to display the preprint in perpetuity. It is made available under aCC-BY 4.0 International license.

Supplementary Figure 1. Six1 is minimally expressed in adipocytes in 7-day vehicle-treated mouse skin.

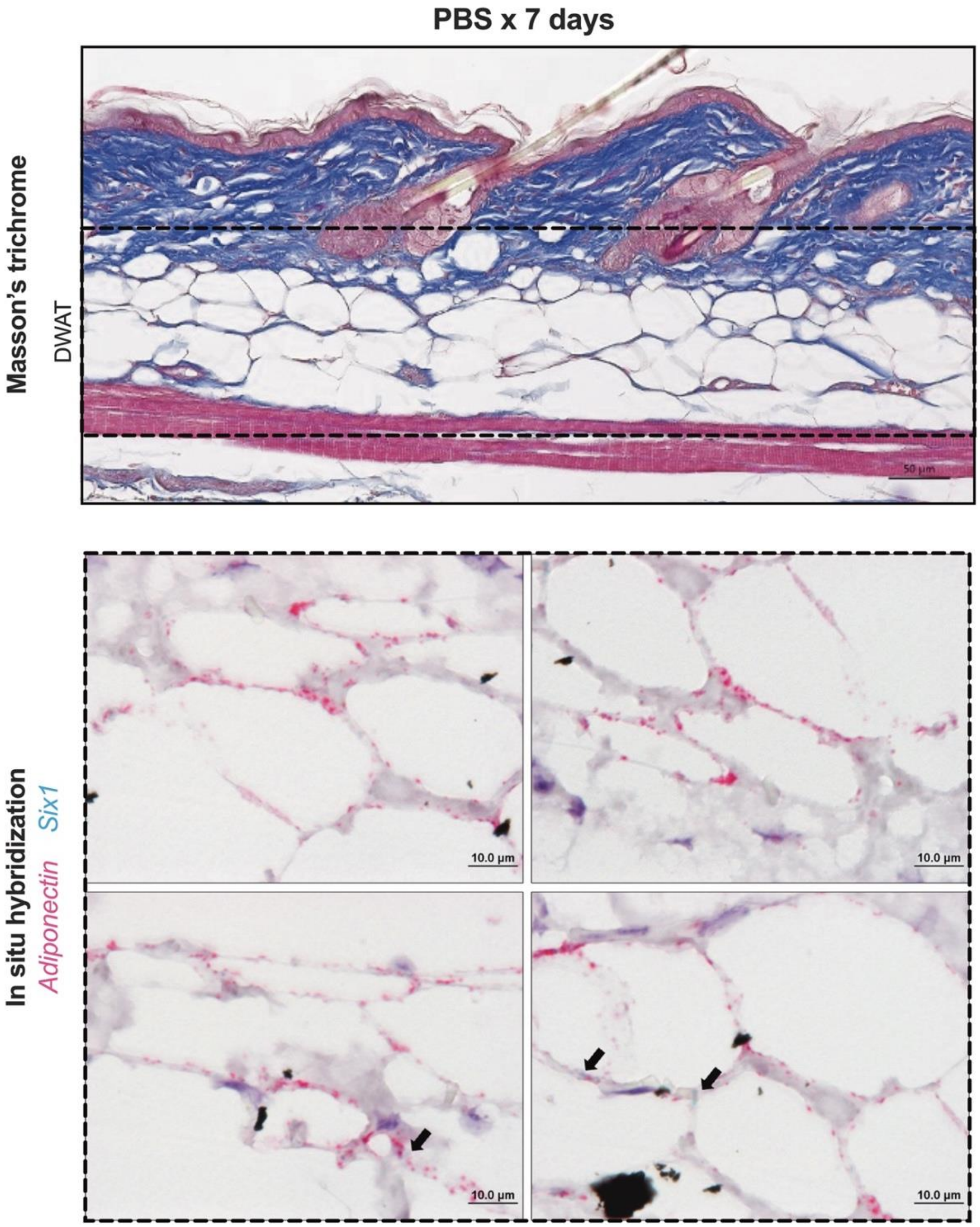

Representative images of mouse dorsal skin injected with SQ vehicle (PBS) for 7 days (n=5). Top: Masson's trichrome staining. DWAT=dermal white adipose tissue. Bottom: Dual *in situ* hybridization for *Adiponectin*/*Adipoq* (pink) and *Six1* (teal). Arrows point to *Six1* signal.

bioRxiv preprint doi: https://doi.org/10.1101/2024.05.22.595271; this version posted May 22, 2024. The copyright holder for this preprint (which was not certified by peer review) is the author/funder, who has granted bioRxiv a license to display the preprint in perpetuity. It is made available under aCC-BY 4.0 International license.

Supplementary Figure 2. siSix1 transfection was effective at days 4 and 6 and minimally changed markers for adipocytes.

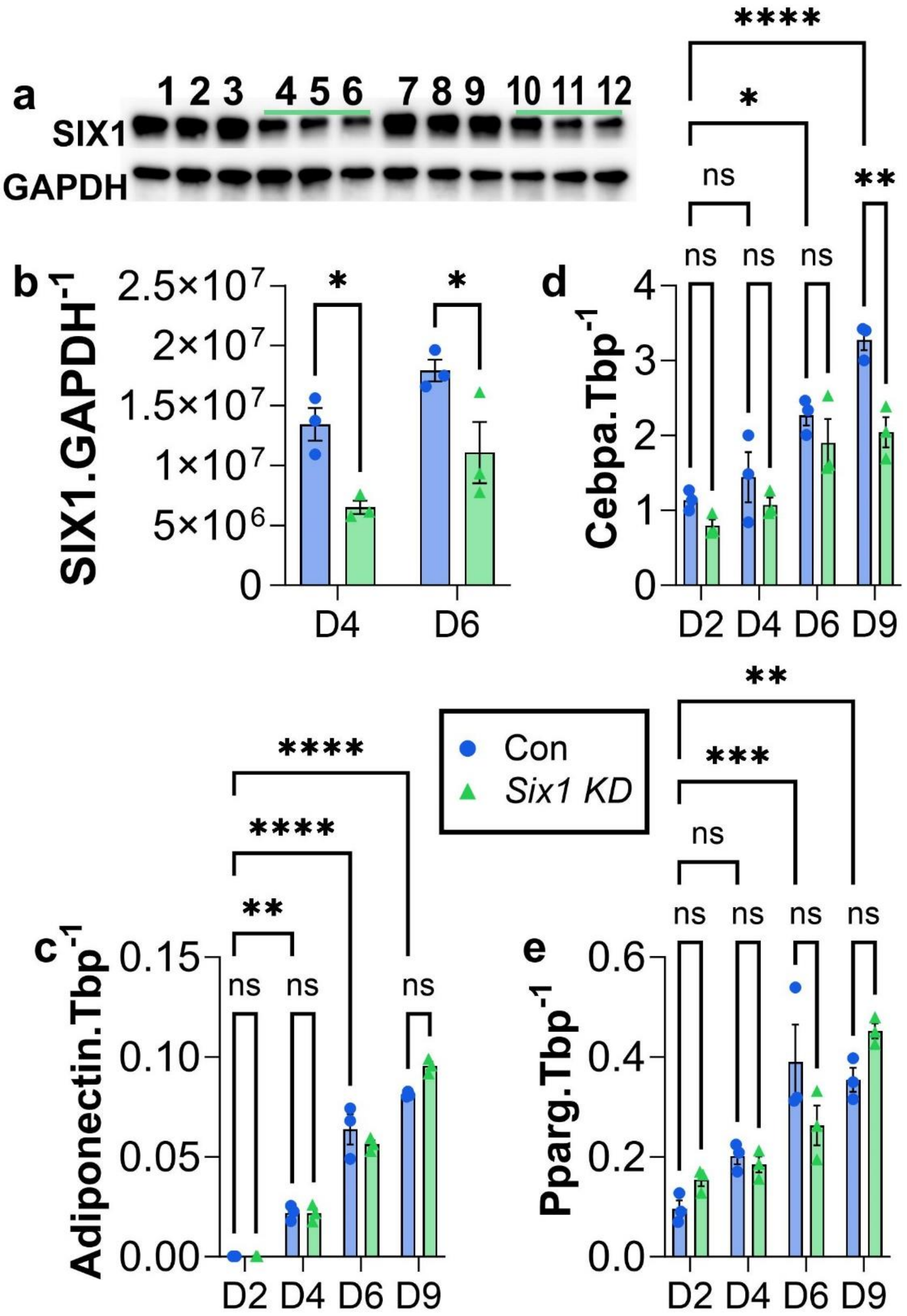

3T3 cells treated with a differentiation cocktail to adipocytes and transfected with either scrRNA (blue bars) or siSix1 (green bars). **a**) western blot for SIX1 or GAPDH lanes 1-3 represent scrRNA transfection on day 4, lanes 4-6 represent siSIX1 transfection on day 4, lanes 7-9 represent scrRNA transfection on day 6 and lanes 10-12 represent siSIX1 transfection on day 6. Expression levels for (**c**) adiponectin, (**d**) Cebpa and (**e**) Pparg from scRNA or siSix1 transfected 3T3 cells on days 2, 4, 6 and 9 after treatment with the differentiation cocktail.

bioRxiv preprint doi: https://doi.org/10.1101/2024.05.22.595271; this version posted May 22, 2024. The copyright holder for this preprint (which was not certified by peer review) is the author/funder, who has granted bioRxiv a license to display the preprint in perpetuity. It is made available under aCC-BY 4.0 International license.

Supplementary Figure 3. Heat maps from nCounter data identifying Serpine1 as a target.

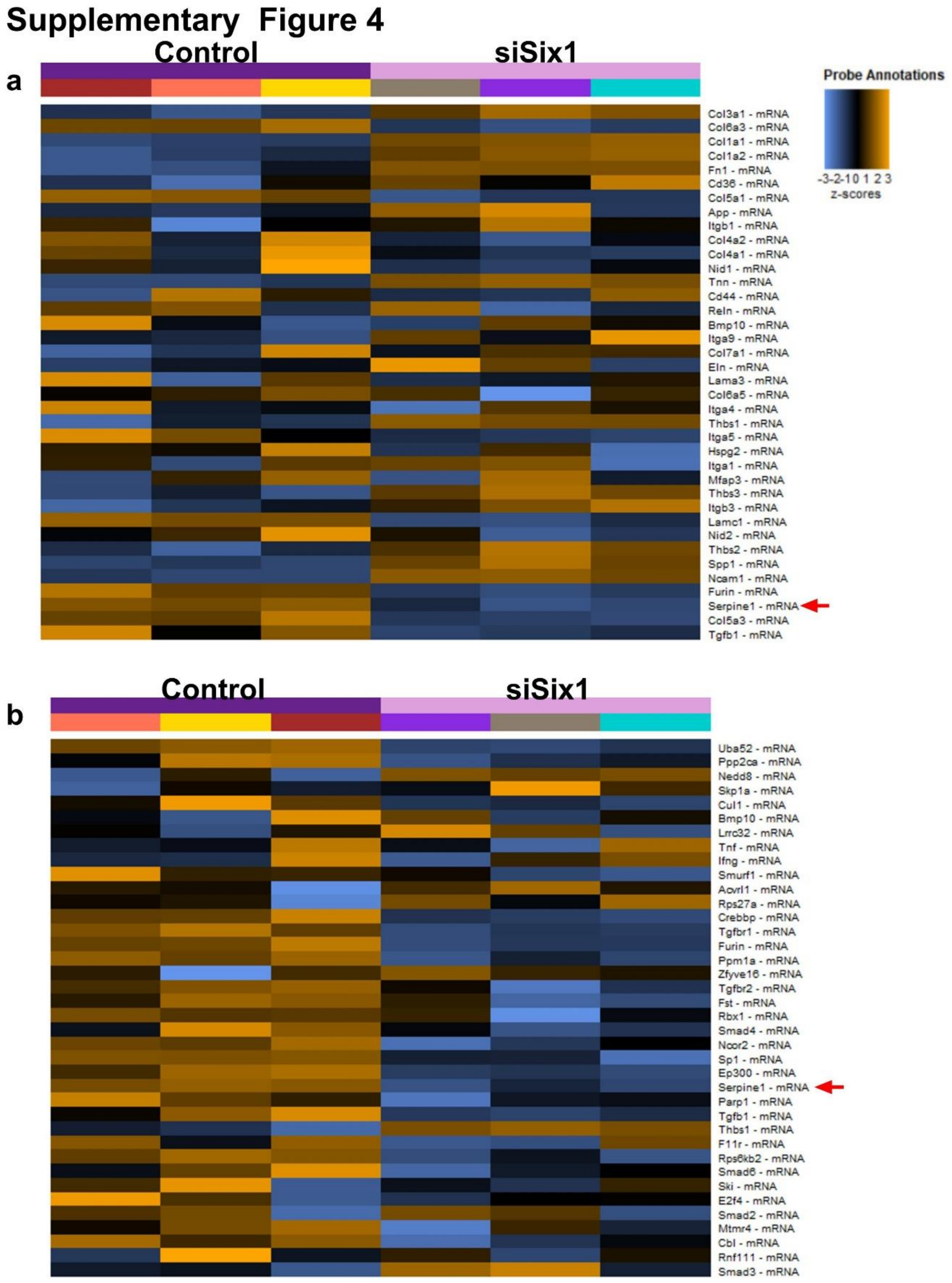

nCounter generated heatmaps for extracellular matrix (ECM) synthesis pathways (**a**) and TGF-β signaling pathway (**b**). Yellow shades represent upregulated genes and blue shades represent downregulated genes. The red arrow points at Serpine1.

bioRxiv preprint doi: https://doi.org/10.1101/2024.05.22.595271; this version posted May 22, 2024. The copyright holder for this preprint (which was not certified by peer review) is the author/funder, who has granted bioRxiv a license to display the preprint in perpetuity. It is made available under aCC-BY 4.0 International license.

Supplementary Figure 4. Volcano plot from nCounter data identifying Serpine1 as a target following SIX1 deletion.

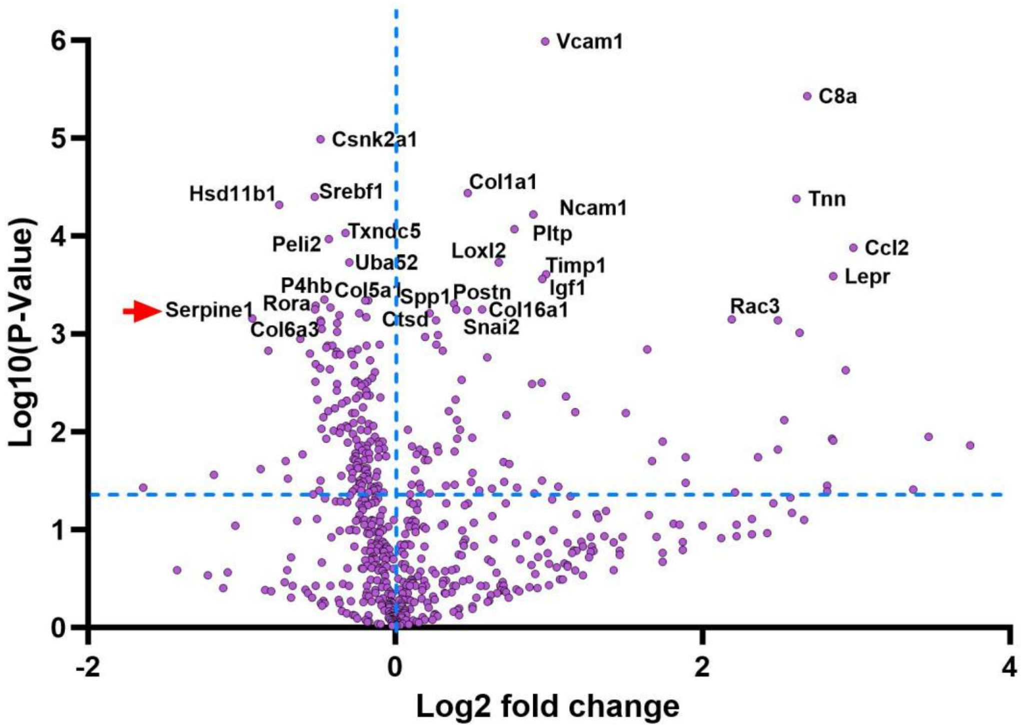

nCounter generated DEG volcano plot from Day 6 scRNA vs siSix1 transfected 3T3 cells treated with a differentiation cocktail to adipocytes. Significant genes are located above the horizontal dotted blue line. The top genes with altered expression are labelled including Serpine 1 identified by a red arrow.

bioRxiv preprint doi: https://doi.org/10.1101/2024.05.22.595271; this version posted May 22, 2024. The copyright holder for this preprint (which was not certified by peer review) is the author/funder, who has granted bioRxiv a license to display the preprint in perpetuity. It is made available under aCC-BY 4.0 International license.

Supplementary Table 1: Mouse SYBR green primers used for quantitative polymerase chain reaction

**Transcript name**	**Forward primer sequence (5' to 3')**	**Reverse primer sequence (3' to 5')**
18s rRNA	GTAACCCGTTGAACCCCATT	CCATCCAATCGGTAGTAGCG
Adiponectin	TGTTCCTCTTAATCCTGCCCA	CCAACCTGCACAAGTTCCCTT
Cebpa	GTCACTGGTCAACTCCAGCAC	CAAGAACAGCAACGAGTACCG
Col1a1	GGTTTCCACGTCTCACCATT	CGGCTCCTGCTCCTCTTAG
Col1a2	AGCAGGTCCTTGGAAACCTT	AAGGAGTTTCATCTGGCCCT
Col6a1	GATGAGGGTGAAGTGGGAGA	CAGCACGAAGAGGATGTCAA
GAPDH	AGGTCGGTGTGAACGGATTTG	TGTAGACCATGTAGTTGAGGTCA
Pparg	GGTGGGCCAGAATGGCATCT	TCTGGGAGATTCTCCTATTGA
Serpine1	TTCAGCCTTGCTTGCCTC	ACACTTTTACTCCGAAGTCGGT
Six1	GAAAGGGAGAACACCGAAAACA	GTGGCCCATATTGCTCTGGA
Tbp	AGAACAATCCAGACTAGCAGCA	GGGAACTTCACATCACAGCTC

bioRxiv preprint doi: https://doi.org/10.1101/2024.05.22.595271; this version posted May 22, 2024. The copyright holder for this preprint (which was not certified by peer review) is the author/funder, who has granted bioRxiv a license to display the preprint in perpetuity. It is made available under aCC-BY 4.0 International license.

Supplementary Table 2: RNAScope probes used for in situ hybridization

Target transcript (RefSeq Accession number)	Target species	Target region	Catalog #
SIX1 (NM_005982.3)	Human	799-2177	ACD; 412401
FABP4 (NM_001442.2)	Human	119 - 826	ACD; 470641-C2
Six1 (NM_009189.3)	Mouse	1932-2819	ACD; 838641
Adiponectin (NM_009605.4)	Mouse	9 - 1233	ACD; 440051-C3

bioRxiv preprint doi: https://doi.org/10.1101/2024.05.22.595271; this version posted May 22, 2024. The copyright holder for this preprint (which was not certified by peer review) is the author/funder, who has granted bioRxiv a license to display the preprint in perpetuity. It is made available under aCC-BY 4.0 International license.

Supplementary Table 3. Top fifty most highly correlated genes with skin *SIX1* in the PRESS cohort by Spearman correlation analysis

Ensembl_ID	Gene symbol	Gene name	*r*	p-value	FDR
ENSG00000166819.10	*PLIN1*	Perilipin 1	0.865	1.30E-19	2.54E-15
ENSG00000187288.9	*CIDEC*	Cell death inducing DFFA like effector C	0.858	5.43E-19	7.07E-15
ENSG00000167676.3	*PLIN4*	Perilipin 4	0.854	1.15E-18	9.13E-15
ENSG00000138207.11	*RBP4*	Retinol binding protein 4	0.854	1.23E-18	9.13E-15
ENSG00000123689.5	*G0S2*	G0/G1 switch gene-2	0.853	1.40E-18	9.13E-15
ENSG00000181092.8	*ADIPOQ*	Adiponectin	0.843	7.82E-18	4.37E-14
ENSG00000165478.6	*HEPACAM*	Hepatic and glial cell adhesion molecule	0.840	1.47E-17	7.19E-14
ENSG00000176485.9	*PLA2G16*	Phospholipase A and acyltransferase 3	0.838	2.01E-17	8.71E-14
ENSG00000079435.8	*LIPE*	Hormone sensitive lipase	0.832	5.58E-17	2.18E-13
ENSG00000184811.3	*TUSC5*	Trafficking regulator of Glut4	0.831	6.58E-17	2.32E-13
ENSG00000175445.13	*LPL*	Lipoprotein lipase	0.830	7.14E-17	2.32E-13
ENSG00000135447.15	*PPP1R1A*	Protein phosphatase 1 regulatory inhibitor subunit 1a	0.819	3.93E-16	1.18E-12
ENSG00000170323.7	*FABP4*	Fatty acid binding protein 4	0.814	9.13E-16	2.55E-12
ENSG00000042286.13	*AIFM2*	Apoptosis inducing factor mitochondria associated 2	0.812	1.15E-15	3.00E-12
ENSG00000185818.7	*NAT8L*	N-acetyltransferase 8 like	0.810	1.61E-15	3.92E-12
ENSG00000174804.3	*FZD4*	Frizzled 4	0.806	2.83E-15	6.51E-12
ENSG00000174697.4	*LEP*	Leptin	0.802	4.51E-15	9.78E-12
ENSG00000129596.4	*CDO1*	Cysteine dioxygenase type 1	0.797	8.75E-15	1.80E-11
ENSG00000173208.3	*ABCD2*	ATP binding cassette subfamily D member 2	0.797	9.23E-15	1.80E-11
ENSG00000005249.11	*PRKAR2B*	Protein kinase camp-dependent type 2 regulatory subunit beta	0.794	1.34E-14	2.49E-11
ENSG00000152270.7	*PDE3B*	Phosphodiesterase 3B	0.794	1.44E-14	2.55E-11
ENSG00000133317.13	*LGALS12*	Galectin 12	0.792	1.71E-14	2.91E-11
ENSG00000168004.8	*PLAAT5*	Phospholipase a and acyltransferase 5	0.792	1.82E-14	2.96E-11
ENSG00000131471.5	*AOC3*	Amine oxidase copper containing 3	0.791	1.90E-14	2.97E-11
ENSG00000134962.6	*KLB*	Klotho beta	0.790	2.20E-14	3.31E-11
ENSG00000165269.11	*AQP7*	Aquaporin 7	0.786	3.88E-14	5.61E-11

bioRxiv preprint doi: https://doi.org/10.1101/2024.05.22.595271; this version posted May 22, 2024. The copyright holder for this preprint (which was not certified by peer review) is the author/funder, who has granted bioRxiv a license to display the preprint in perpetuity. It is made available under aCC-BY 4.0 International license.

ENSG00000164638.9	*SLC29A4*	Solute carrier family 29 member 4	0.785	4.13E-14	5.76E-11
ENSG00000119927.12	*GPAM*	Glycerol-3-phosphate acyltransferase, mitochondrial	0.780	7.65E-14	1.03E-10
ENSG00000169692.11	*AGPAT2*	1-acylglycerol-3-phosphate o-acyltransferase 2	0.777	1.15E-13	1.49E-10
ENSG00000198624.11	*CCDC69*	Coiled-coil domain containing 69	0.773	1.81E-13	2.28E-10
ENSG00000149124.9	*GLYAT*	Glycine-N-acyltransferase	0.772	2.03E-13	2.48E-10
ENSG00000221968.7	*FADS3*	Fatty acid desaturase 3	0.771	2.30E-13	2.72E-10
ENSG00000056998.17	*GYG2*	Glycogenin 2	0.769	2.88E-13	3.31E-10
ENSG00000158571.9	*PFKFB1*	6-Phosphofructo-2-Kinase/Fructose-2,6-Biphosphatase 1	0.768	3.17E-13	3.54E-10
ENSG00000076706.13	*MCAM*	Melanoma cell adhesion molecule	0.763	5.59E-13	6.06E-10
ENSG00000234840.1	*LINC01239*	Long intergenic non-protein coding rna 1239	0.759	8.51E-13	8.89E-10
ENSG00000129675.14	*ARHGEF6*	Rac/cdc42 guanine nucleotide exchange factor 6	0.759	8.65E-13	8.89E-10
ENSG00000123612.14	*ACVR1C*	Activin A receptor type 1c	0.753	1.61E-12	1.61E-09
ENSG00000124253.10	*PCK1*	Phosphoenolpyruvate Carboxykinase 1	0.752	1.79E-12	1.75E-09
ENSG00000009950.14	*MLXIPL*	Mlx interacting protein like	0.747	3.18E-12	3.03E-09
ENSG00000281769.1	*LINC01239*	Long intergenic non-protein coding rna 1239	0.730	1.64E-11	1.52E-08
ENSG00000189367.13	*KIAA0408*	Uncharacterized protein KIAA0408	0.729	1.86E-11	1.68E-08
ENSG00000177666.14	*ATGL*	Adipose triglyceride lipase	0.729	1.89E-11	1.68E-08
ENSG00000158186.11	*MRAS*	Muscle RAS oncogene homolog	0.727	2.24E-11	1.94E-08
ENSG00000277737.2	*FP325317.1*	*uncharacterized transcript*	0.726	2.40E-11	2.04E-08
ENSG00000120049.17	*KCNIP2*	Potassium voltage-gated channel interacting orotein 2	0.722	3.49E-11	2.87E-08
ENSG00000186205.11	*MTARC1*	Mitochondrial amidoxime reducing component 1	0.722	3.53E-11	2.87E-08
ENSG00000171914.13	*TLN2*	Talin 2	0.722	3.66E-11	2.92E-08
ENSG00000119729.9	*RHOQ*	Ras homolog family member Q	0.717	5.67E-11	4.43E-08
ENSG00000151632.15	*AKR1C2*	Aldo-keto reductase family 1 member c2	0.716	5.99E-11	4.59E-08

bioRxiv preprint doi: https://doi.org/10.1101/2024.05.22.595271; this version posted May 22, 2024. The copyright holder for this preprint (which was not certified by peer review) is the author/funder, who has granted bioRxiv a license to display the preprint in perpetuity. It is made available under aCC-BY 4.0 International license.

Supplementary Table 4. Demographic and clinical features of biopsied participants in Supplementary Figure 1.

Panel	Age (yrs)	Sex	Race	Disease subtype	Disease duration (yrs)	mRSS	local skin score
a	54	Female	Caucasian	Control	N/A	N/A	N/A
b	45	Female	Caucasian	dcSSc	2.3	22	2
c	62	Female	Caucasian	dcSSc	3.4	32	2
d	45	Female	Caucasian	dcSSc	5.0	20	2

Age, disease duration, mRSS, and local skin score were recorded at the time of biopsy. Local skin scores were taken adjacent to the site of the biopsy.
